# Tracheal and Cricotracheal Resection With End-to-End Anastomosis for Locally Advanced Thyroid Cancer: A Systematic Review of the Literature on 656 Patients

**DOI:** 10.3389/fendo.2021.779999

**Published:** 2021-11-11

**Authors:** Cesare Piazza, Davide Lancini, Michele Tomasoni, Anil D’Cruz, Dana M. Hartl, Luiz P. Kowalski, Gregory W. Randolph, Alessandra Rinaldo, Jatin P. Shah, Ashok R. Shaha, Ricard Simo, Vincent Vander Poorten, Mark Zafereo, Alfio Ferlito

**Affiliations:** ^1^ Unit of Otorhinolaryngology – Head and Neck Surgery, Azienda Socio Sanitaria Territoriale (ASST) Spedali Civili of Brescia, Brescia, Italy; ^2^ Department of Medical, Surgical and Radiological Sciences and Public Health, School of Medicine, University of Brescia, Brescia, Italy; ^3^ Director Oncology Apollo Group of Hospitals, Mumbai, India; ^4^ Department of Head and Neck Oncology, Gustave Roussy, Université Paris Saclay, Paris, France; ^5^ Department of Head and Neck Surgery, University of Sao Paulo Medical School and Antonio Cândido (AC) Camargo Cancer Center, Sao Paulo, Brazil; ^6^ John and Claire Bertucci Endowed Chair in Thyroid Surgical Oncology, Harvard Medical School, Boston, MA, United States; ^7^ University of Udine School of Medicine, Udine, Italy; ^8^ Department of Surgery, Head and Neck Service, Memorial Sloan Kettering Cancer Center, New York, NY, United States; ^9^ Department of Oncology, Radiotherapy and Plastic Surgery, Sechenov University, Moscow, Russia; ^10^ Jatin P Shah Chair in Head and Neck Surgery, Memorial Sloan Kettering Cancer Center, New York, NY, United States; ^11^ Department of Otorhinolaryngology – Head and Neck Surgery, Head, Neck and Thyroid Oncology Unit, Guy’s and St Thomas’ Hospital National Health Service (NHS) Foundation Trust and King’s College London, London, United Kingdom; ^12^ Otorhinolaryngology – Head and Neck Surgery, University Hospitals Leuven, Leuven, Belgium; ^13^ Department of Oncology, Section Head and Neck Oncology, Katholieke Universiteit (KU) Leuven, Leuven, Belgium; ^14^ Division of Surgery, Department of Head and Neck Surgery, The University of Texas MD Anderson Cancer Center, Houston, TX, United States; ^15^ Coordinator of the International Head and Neck Scientific Group, Padua, Italy

**Keywords:** thyroid cancer, airway, surgery, tracheal resection, crico-tracheal resection

## Abstract

Airway involvement by advanced thyroid carcinoma (TC) constitutes a negative prognosticator, besides being a critical clinical issue since it represents one of the most frequent causes of death in locally advanced disease. It is generally agreed that, for appropriate laryngo-tracheal patterns of invasion, (crico-)tracheal resection and primary anastomosis [(C)TRA] is the preferred surgical technique in this clinical scenario. However, the results of long-term outcomes of (C)TRA are scarce in the literature, due to the rarity of such cases. The relative paucity of data prompts careful review of the available relevant series in order to critically evaluate this surgical technique from the oncologic and functional points of view. A systematic review was conducted according to the Preferred Reporting Items for Systematic Reviews and Meta-Analyses statement on the PubMed, Scopus, and Web of Science databases. English-language surgical series published between January 1985 and August 2021, reporting data on ≥5 patients treated for TC infiltrating the airway by (C)TRA were included. Oncologic outcomes, mortality, complications, and tracheotomy-dependency rates were assessed. Pooled proportion estimates were elaborated for each end-point. Thirty-seven studies were included, encompassing a total of 656 patients. Pooled risk of perioperative mortality was 2.0%. Surgical complications were reported in 27.0% of patients, with uni- or bilateral recurrent laryngeal nerve palsy being the most common. Permanent tracheotomy was required in 4.0% of patients. Oncologic outcomes varied among different series with 5- and 10-year overall survival rates ranging from 61% to 100% and 42.1% to 78.1%, respectively. Five- and 10-year disease specific survival rates ranged from 75.8% to 90% and 54.5% to 62.9%, respectively. Therefore, locally advanced TC with airway invasion treated with (C)TRA provides acceptable oncologic outcomes associated with a low permanent tracheotomy rate. The reported incidence of complications, however, indicates the need for judicious patient selection, meticulous surgical technique, and careful postoperative management.

## Introduction

Advanced resectable (T4a) thyroid cancer (TC) is a relatively uncommon clinical scenario, especially when dealing with differentiated tumors, being reported in just 5-15% of papillary carcinomas (1–4). This condition is associated with a significantly lower long-term survival rate compared to early-stage disease (1, 5, 6), particularly when the macroscopic extra-thyroidal extension involves more than one adjacent anatomical structure (7).

Aerodigestive tract invasion is more often seen in locally recurrent differentiated thyroid carcinoma (DTC) than at initial presentation. On the other hand, 60-70% of patients with such advanced neoplasms will have poorly differentiated or anaplastic carcinomas (8). The most frequently involved neighboring structures (after the strap muscles and recurrent laryngeal nerves [RLN]) are the upper trachea and laryngo-tracheal junction, due to their anatomic contiguity and relationship with the thyroid gland (9, 10), with a reported incidence of invasion of 0.4-0.7% of all TC (11). In descending order of frequency, the fourth and fifth most affected structures are the pharyngo-esophageal conduit and major vessels in the neck (8–10). The source of aerodigestive tract involvement is most frequently the primary tumor, while metastatic lymph nodes are responsible for less than 20% of cases (8).

Airway invasion by TC typically occurs in men (twice more frequently than in females), with a peak incidence in the sixth decade (11) and, usually, involves tumors larger than 3.7 cm (3, 12). Although rare, airway invasion has also been reported in the younger age group, considered to be in the “low-risk” prognostic category (13). Uncontrolled tumor progression in the airway represents one of the most frequent causes of death for TC, especially in the presence of unresectable tumors or loco-regional disease in which complete resection was not achieved (6, 14–16). Thus, in order to increase the chance of cure of these advanced neoplasms invading the airway, the first goal is to achieve a R0 resection within negative margins (5, 17, 18). However, due to the relative paucity of large series on this topic and in the absence of any prospective trials, the indications and comparative outcomes of different surgical techniques for airway management in advanced TC are still a matter of debate. There is general agreement that shaving the tumor off from the laryngo-tracheal axis is acceptable when the lesion involves only its external perichondrium (Shin I according to the classification by Shin et al.) (19), but there is no consensus on the best surgical technique for more extensive tumors (infiltrating the full-thickness of the cartilage [Shin II] or through it into the submucosa [Shin III] or the tracheal lumen [Shin IV]). Essentially, there are two different schools of thought: on one side, window resection with primary or secondary closure of the airway gap by soft tissue local flaps (20, 21) and, on the other, circumferential (crico-)tracheal resection with primary end-to-end anastomosis ([C]TRA) (22). Other groups have tried to design comprehensive, but somewhat cumbersome, algorithms in which both procedures can be performed according to the site, length, and width of airway involvement (8, 23). Head-to-head oncologic comparisons between these two surgical approaches are seriously limited by the low incidence of this condition, the heterogeneity of patients treated, and significant selection biases due to the retrospective nature of the studies. On the other hand, it is possible to objectively analyze postoperative morbidity, complication rates, and quality of life reported in the literature for each type of surgical technique.

The aim of this systematic review was to collect all the available English-language surgical series published between January 1985 and August 2021, reporting data on ≥5 patients treated for TC infiltrating the airway by (C)TRA, to better understand oncologic outcomes, complication rates, and airway-related quality of life.

## Materials and Methods

### Article Collection

A systematic review of the literature was conducted according to the Preferred Reporting Items for Systematic Reviews and Meta-Analyses (PRISMA) statement (24). The search was simultaneously conducted on the PubMed, Scopus, and Web of Science online databases, and updated to August 16, 2021. In order to retrieve all the publications dealing with (C)TRA for laryngo-tracheal involvement by TC the query string was composed as (tracheal resection) OR (tracheal involvement) AND (thyroid cancer) OR (thyroid neoplasm) OR (thyroid tumor). The search was conducted by two authors (C.P. and D.L.) who independently assessed the eligibility of the studies by screening article titles and abstracts, and then discussed their inclusion by reading the full-text of the selected works. Discrepancies were clarified by discussion between authors.

### Eligibility Criteria

The Population/problem Intervention/exposure Comparison, Outcome, and Study design (PICOS) model was adopted for the review (25) (**Table 1**). The inclusion criteria were as follows: English language, publication from January 1, 1985 to the last day of online search (August 16, 2021), articles including data on (C)TRA for airway involvement by TC and reporting a case series of at least 5 patients. Exclusion criteria were: case reports, case series with less than 5 patients, papers purely describing results of surgical techniques different from (C)TRA (e.g., shaving, window resections, total laryngectomy) or that did not report sufficient data on outcomes and complications and focused on other related issues (e.g., radiological or clinical diagnosis, anesthetic issues, adjuvant treatments). Additionally, papers with duplicated or overlapping data from the same center were excluded, maintaining, when possible, the largest and more recent study among those available. Finally, a case series published by the first author (C.P.) (26), already included in this systematic review, was updated with data of patients treated from the time of the article publication (2016) to date, and their oncologic outcomes updated accordingly.

**Table 1:** PICOS model for the present systematic review.

P (population)	656 patients from 37 studies adhering to the inclusion criteria detailed in Materials and Methods
I (intervention)	(Crico-)tracheal resection and anastomosis for thyroid cancer invading the airway
C (comparator)	No comparison was intentionally performed with other surgical techniques and/or treatment modalities
O (outcomes)	Perioperative mortality, complication, postoperative tracheostomy-dependency rates, and oncologic outcomes
S (study design)	Systematic review

### Quality Assessment

For each paper included in the systematic review, at the end of the selection process, evaluation of its quality was carried out following the Newcastle-Ottawa Scale (NOS) adapted for cross-sectional, cohort and case-control studies (27). The NOS was considered the evaluation method of choice, based on the recent literature (28, 29). The quality assessment was independently estimated by two different authors (D.L. and M.T.).

### Data Collection and Statistical Analysis

Data on study design, number of patients, age, gender, diagnostic work-up, TC histology and degree of airway invasion, length and type of resection, perioperative mortality, surgical complications, rate of patients who remained tracheostomy-dependent after (C)TRA, and oncological outcomes were collected, and a specific database was built.

The primary outcome was proportion of patients who developed a complication, calculated as the number of patients with reported complications divided by the total number of patients treated by (C)TRA for TC. Secondary outcome was the proportion of tracheostomy-dependent patients, defined as the number of patients with long-term tracheostomy dependency divided by the total number of patients treated by (C)TRA.

Meta-analysis of proportions was conducted through a generalized linear mixed model based on logit transformation (30). Pooled analyses are presented as forest plots. For each study, proportions and relative 95% confidence interval (CI) are depicted as gray squares and horizontal lines, respectively. The weight of each study on the overall effect estimate is reported and represented by the square size. The pooled proportion estimate and relative 95% CI, depicted as a diamond, are reported at the bottom of the forest plot. Heterogeneity between studies was assessed with Higgins I^2^ and τ^2^ tests (31), defined as low if I^2^<25%, moderate if between 25-50%, and substantial if >50% (32).

Publication bias was assessed through funnel plot assessment. Statistical analysis was performed with R (version 4.0.5, R foundation for Statistical Computing, Vienna, Austria). Statistical significance was defined as p<0.05.

## Results

### Article Collection

**Figure 1:**
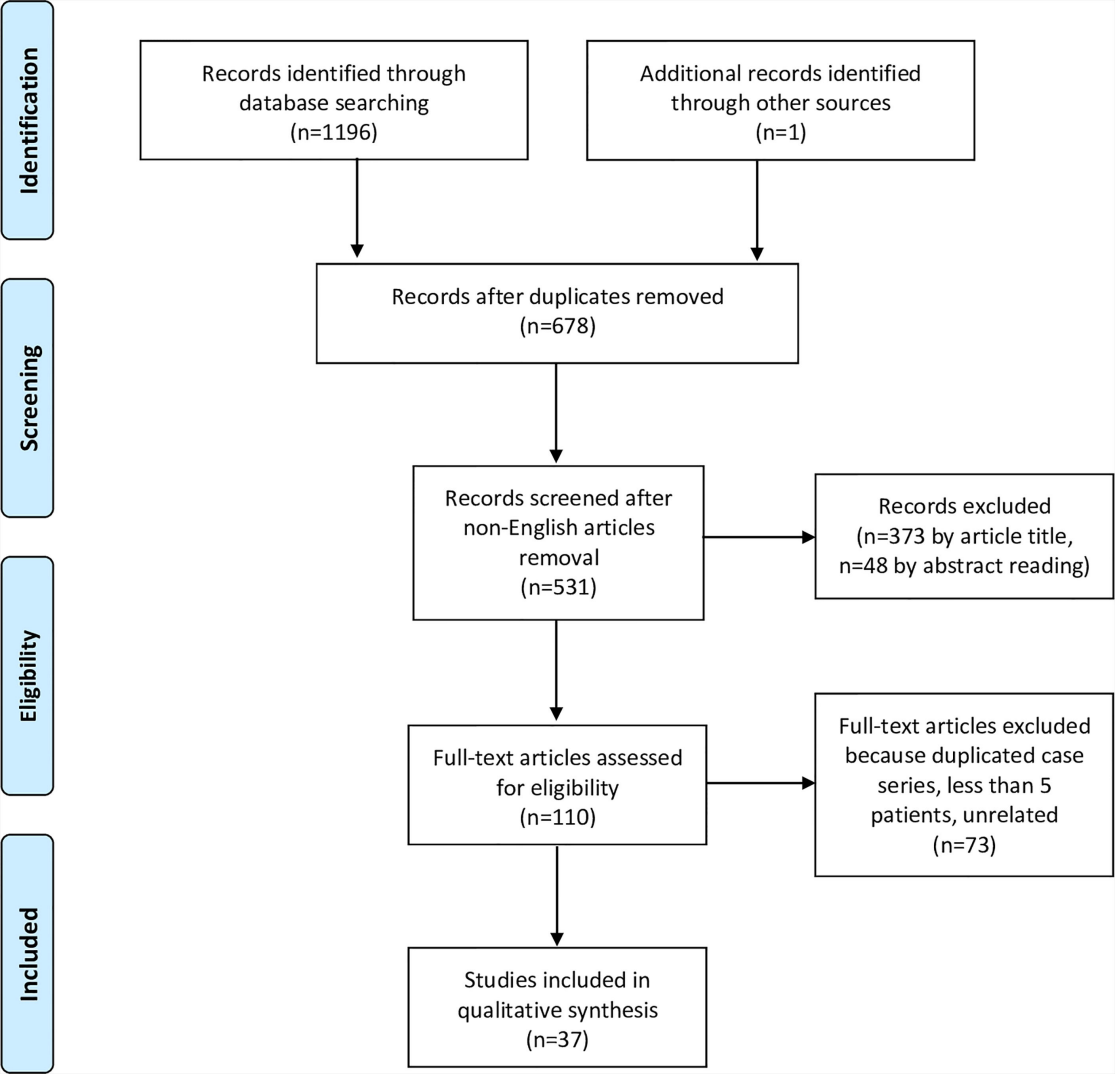
Flowchart showing the study selection process according to the Preferred Reporting Items for Systematic Reviews and Meta-Analyses (PRISMA) statement.

The initial literature search yielded 1196 titles (525 records came from the PubMed database, 407 from Scopus, and 264 from Web of Science). Among these, 519 articles were excluded because present in two databases, and 147 due to publication in a language other than English. One article (33) was added from other sources, after being identified through the references of other manuscripts. Three-hundred-seventy-three articles were excluded after review of the title, and 48 by the abstract. From the remaining 110 full-text articles, 73 were excluded because they did not meet the eligibility criteria. Finally, 37 papers (3, 26, 33–67) were considered appropriate for the present systematic review (**Figure 1**, **Table 2**).

**Table 2:** Studies included in the systematic review of English-language, non-overlapping, surgical series including ≥5 patients treated by (C)TRA for TC invading the airway between January 1985 and August 2021 (No. of series=37, No. of patients=656). The table reports details about TC histotype, mortality, complications, tracheostomy-dependency, and oncologic outcomes.

Study no.	Author (Institution)	Year	No. of pts.	Histotypes	Perioperative mortality rate	Complication rate	Tracheostomy-dependency rate	Oncologic outcomes
1	Tovi et al. (33) (Soroka University, Beer-Sheva, Israel)	1985	5	DTC	0%	NA	0%	NA
2	Fujimoto et al. (34) (Tokyo Women’s Medical College Hospital, Tokyo, Japan)	1986	6	DTC	0%	17% (permanent hypoparathyroidism x1)	0%	100% alive and well, 18-53 mos
3	Ishihara et al. (35) (Keyo University, Tokyo, Japan)	1991	60	Mixed	0%	47% (bilateral RLN palsy x21, temporary dysphagia x3, hypoparathyroidism x2, anastomotic stenosis x2, pharyngeal stenosis x1, vocal fold edema x1)	13% (permanent tracheotomy for bilateral RLN palsy x8)	10-yr OS 78.1% (in 34 R0 pts)
								10-yr OS 22% (in 26 R1-R2 pts)
4	Maeda (36) (Kakawa Medical School, Kagawa, Japan)	1993	44	Mixed	0%	2% (bilateral RLN palsy x1)	NA	NA
5	Ozaki et al. (37) (Ito Hospital, Tokyo, Japan)	1995	21	Mixed	0%	5% (bilateral RLN palsy x1)	5% (permanent tracheotomy for bilateral RLN palsy x1)	76% alive and well, 17-121 mos
								19% alive with distant metastases, 21-99 mos
								5% dead for unrelated causes at 12 mos
6	Zannini et al. (38) (San Raffaele Hospital, University of Milan, Italy)	1996	8	DTC	0%	25% (anastomotic granulomas x2)	0%	50% alive and well, 11-109 mos
								25% alive with distant metastases, 75-127 mos
								25% dead for distant or regional disease, 26-32 mos
7	Musholt et al. (39) (Hannover University Medical School, Hannover, Germany)	1999	11	Mixed	9% (prolonged assisted ventilation with multiorgan failure x1)	18% (aspiration pneumonia x1, prolonged ICU treatment x1)	0%	64% alive and well, 3-43 mos
								18% dead for local or distant disease, 8-25 mos
								9% dead for unrelated causes, 42 mos
8	Yang et al. (40) (Veterans General Hospital – Taipei and National Yang-Ming University, Taipei, Taiwan)	2000	8	DTC	0%	12% (anastomotic leak x1)	0%	62% alive and well, 14-183 mos
								38% alive with local, regional or distant disease, 39-71 mos
9	Koike et al. (41) (Noguchi Thyroid Clinic and Hospital Foundation, Oita, Japan)	2001	7	DTC	0%	NA	NA	100% alive and well, 15-22 mos
10	Kato et al. (42) (St. Marianna, Kawasaki and Yamagata University School of Medicine, Yamagata, Japan)	2003	18	Mixed	0%	5% (unilateral RLN palsy x1)	0%	NA
11	Nakao et al. (43) (Osaka Police Hospital, Osaka, Japan)	2004	40	DTC	7% (anastomotic dehiscence and fatal bleeding x2, anastomotic dehiscence and mediastinitis x1)	30% (anastomotic dehiscence x4, unilateral RLN palsy x3, pneumonia x3, bilateral RLN palsy x1, acute myocardial infarction x1)	7% (bilateral RLN palsy x3)	10-yr OS 67.7%
12	Tsai et al. (44) (National Cheng Kung University Hospital and Chi-Mei Hospital, Taiwan, China)	2005	16	DTC	6% (anastomotic dehiscence and fatal bleeding x1)	25% (anastomotic dehiscence x2, anastomotic granulomas x2)	0%	5-yr OS 88%
13	Wada et al. (45) (Yokohama City University Medical Center, Kanagawa, Japan)	2006	5	DTC	0%	20% (bilateral RLN palsy x1)	20% (bilateral RLN palsy x1)	5-yr DSS 83.9%
								10-yr DSS 62.9%
14	Segal et al. (3) (Rabin Medical Center, Petah Tiqva, Israel)	2006	6	DTC	0%	NA	NA	5-yr OS 75%
15	Gaissert et al. (46) (Massachusetts General Hospital, Boston, Massachusetts)	2007	69	Mixed	1% (glottic edema with fatal respiratory insufficiency x1)	61% (temporary tracheostomy x13, other complications x7, bilateral RLN palsy x6, permanent hypoparathyroidism x5, aspiration x5, anastomotic dehiscence x3, dysphagia x3)	4% (bilateral RLN palsy x2, anastomotic dehiscence x1)	15-yr OS 26% (in DTC pts)
								15-yr DFS 22% (in DTC pts)
16	Brauckhoff et al. (47) (University Hospital Halle, Saale, Germany)	2010	16	Mixed	6% (fatal anastomotic dehiscence x1)	31% (anastomotic dehiscence x2, esophageal fistula x2, other x1)	0%	5-yr DSS 85.1%
								10-yr DSS 73.8%
17	Mutrie et al. (48) (Emory University School of Medicine, Atlanta, Georgia)	2011	9	Mixed	0%	NA	0%	5-yr OS 80%
18	Shadmehr et al. (49) (Shahid Beheshti University of Medical Sciences, Tehran, Iran)	2012	18	Mixed	4% (anastomotic dehiscence and fatal mediastinitis x1)	30% (anastomotic dehiscence x2, unilateral RLN palsy x2, permanent hypoparathyroidism x1, temporary tracheostomy x1)	0%	5-yr OS 61% (immediate resection group)*
								5-yr OS 28% (delayed resection group)*
19	Ch’ng et al. (50) (Sidney Head and Neck Cancer Institute, Australia)	2012	6	Mixed	0%	0%	33%^§^ (bilateral RLN palsy x2)	NA
20	Mossetti et al. (51) (Ospedale Molinette, University of Turin, Italy)	2013	8	Mixed	0%	62% (transient hypoparathyroidism x3, anastomotic leak x2, bleeding x1)	0%	12% DOD, 30 mos
								63% AWD, 3-67 mos
								25% NED, 10-25 mos
21	Morisod et al. (52) (University Hospital CHUV, Lausanne, Switzerland)	2014	6	Mixed	17% (massive anastomotic dehiscence with tracheo-innominate fistula x1)	50% (minor tracheal dehiscence x1, pneumonia x1, SIADH x1)	0%	17% DOC, perioperative death
								17% DOD, 2 mos
								66% NED, 6-41 mos
22	Lin et al. (53) (Memorial Hospital of Sun Yat-sen University, Guangzhou, China)	2014	19	DTC	5% (esophageal fistula x1)	26% (bilateral RLN palsy x2, esophageal fistula x2, anastomotic dehiscence x2, anastomotic stenosis x1)	16% (bilateral RLN palsy x1, anastomotic dehiscence x2)	5% DOC, 3 mos
								10% AWD, 11-30 mos
								85% NED, 2-55 mos
23	Hartl et al. (54) (Institute Gustave Roussy, Paris, France)	2014	23	Mixed	NA	NA	NA	5- and 10-yr OS 73% and 59%°
								5- and 10-yr LC 83% (100% for R0 and 75% for R1)°
								5- and 10-yr DSS 89% (95% for R0 and 84% for R1)°
24	Ranganath et al. (55) (Kidwai Memorial Institute of Oncology, Bengaluru, India)^£^	2015	10	Mixed	10% (chyle leak and septicaemia x1)	70% (hypoparathyroidism x7, aspiration x1)	0%	100% alive and well, 3-24 mos
25	Peng et al. (56) (The Second Xiangya Hospital of Central South University, Changsha, China)	2015	14	Mixed	0%	14% (anastomotic dehiscence x1, tracheomalacia x1)	7% (anastomotic dehiscence x1)	NA
26	Pappalardo et al. (57) (University of Insubria, Varese, Italy)	2016	7	Mixed	0%	0%	0%	100% NED, 18-108 mos
27	Kim et al. (58) (Seoul National University Hospital, Seoul, Republic of Korea)	2016	37	DTC	0%	NA	NA	5-yr DSS 90%
								10-yr DSS 85%
28	Avenia et al. (59) (Santa Maria Hospital, University of Perugia, Italy)	2016	28	DTC	0%	32% (hypoparathyroidism x3, aspiration x2, dysphagia x1, anastomotic dehiscence x2, bilateral RLN palsy x1)	7% (bilateral RLN palsy x1, anastomotic dehiscence x1)	5-yr OS 70%*
29	Su et al. (60) (MD Anderson Cancer Institute. Houston, Texas)	2016	7	DTC	0%	NA	NA	NA
30	Wang et al. (61) (Memorial Sloan Kettering Cancer Center, New York, New York)	2016	7	DTC	0%	28% (temporary tracheotomy x2)	0%	NA
31	Piazza et al.^#^ (26) (Spedali Civili, University of Brescia, Italy)	2016	33	Mixed	0%	28% (anastomotic dehiscence x3, unilateral RLN palsy x2, bilateral RLN palsy x1, bleeding x1, pulmonary embolism x1, pneumonia x1)	3% (bilateral RLN palsy x1)	5-yr OS (entire series) 63.4%
								10-yr OS (entire series) 42.1%
								5-yr OS DTC 81.8%
								10-yr OS DTC 52.2%
								5-yr OS non-DTC 12.5%
								10-yr OS non-DTC 12.5%
								5-yr DSS (entire series) 75.8%
								10-yr DSS (entire series) 54.5%
								5-yr DSS DTC 86.1%
								10-yr DSS DTC 59.9%
								5-yr DSS non-DTC 50%
								5-yr DSS non-DTC 50%
32	Chen et al. (62) (Shandong Cancer Hospital, Jinan, China)	2017	21	DTC	0%	43% (temporary dysphagia x11, temporary hypoparathyroidism x9, air leak x5)	0%	5-yr OS 100%
								5% DOC, 72 mos
								5% DOD, 74 mos
								28% AWD, 19-61 mos
								62% NED, 8-78 mos
33	Gupta et al. (63) (Basavatarakam Indo American Cancer Hospital & Research Institute, Hyderabad, India)	2020	11	DTC	0%	64% (temporary hypoparathyroidism x7, temporary tracheotomy x1)	0%	81.2% OS (median follow-up 41 mos)
34	Chen et al. (64) (Sichuan Cancer Hospital, Chengdu, China)	2020	5	Mixed	0%	NA	NA	100% alive and well, 24-40 mos
35	Tiwari et al. (65) (Chennai Cancer Institute, Tamil Nadu, India)	2020	23	Mixed	0%	39% (air leak x5, bleeding x2, anastomotic dehiscence x1, aspiration x1)	0%	5-yr OS° 81.7%
								10-yr OS° 47.8%
								15-yr OS° 35.9%
36	Sharanappa et al. (66) (Sanjay Gandhi Postgraduate Institute of Medical Sciences, Lucknow, India)	2021	5	DTC	0%	20% (bleeding x1)	20% (bilateral RLN palsy x1)	5-yr OS 80%
37	Chakravarthy et al. (67) (Christian Medical College, Vellore, Tamil Nadu, India)	2021	19	Mixed	0%	36%° (temporary tracheotomy x1, temporary hypoparathyroidism x6, permanent hypoparathytoidism x1)	11% (bilateral RLN palsy x2)	13.6% DOD°
								36.4% AWD°
								13.6% NED°

DTC, differentiated thyroid cancer; NA, not available; RLN, recurrent laryngeal nerve; OS, overall survival; DSS, disease specific survival; R0, microscopically free surgical margins; R1, microscopically involved surgical margins; R2, macroscopically involved surgical margins; NED, no evidence of disease; DOD, dead of disease; AWD, alive with disease; ^£^includes partial data from overlapping series from Shenoy et al. (2012); *OS has been calculated without distinction between TRA/CTRA patients and laryngectomees; ^§^in 2 pts. both RLN were intentionally sacrificed carrying to permanent tracheostomy; °data non distinguishing between TRA/CTRA procedures and other types of airway surgeries; ^#^this series has been adjourned at August 2021.

### Quality Assessment

According to the NOS adapted for cross-sectional studies (range of the scale, 0-9), the scores ranged from 2 to 7 (median, 5). Detailed scores for each article are reported in **Table 3**. All included manuscripts were retrospective single institution cross-sectional studies, except for one (42), which was a retrospective bi-institutional case series.

**Table 3:** Quality assessment of papers included in the present systematic review (N=37).

Source	Selection				Comparability	Outcome			Total
	Representativeness of the exposed cohort	Selection of the non exposed cohort	Ascertainment of exposure	Demonstration that outcome of interest was not present at start of study	Comparability based on design and analysis	Assesment of outcome	Was follow-up long enough for outcomes to occur	Adequacy of follow up of cohorts	
Avenia et al. (59)	*	*	*			*			4
Brauckhoff et al. (47)	*	*	*		*	*	*	*	7
Chakravarthy et al. (67)	*		*			*	*	*	5
Chen et al. (62)	*	*	*		*	*	*	*	7
Chen et al. (64)			*			*			2
Ch’ng et al. (50)	*	*	*			*		*	5
Fujimoto et al. (34)	*	*	*		*	*		*	6
Gaissert et al. (46)	*	*	*		*	*	*	*	7
Gupta et al. (63)	*	*	*		*	*	*	*	7
Hartl et al. (54)	*	*	*			*	*	*	6
Ishihara et al. (35)	*		*			*	*	*	5
Kato et al. (42)	*		*			*			3
Kim et al. (58)	*	*	*			*	*	*	6
Koike et al. (41)	*	*	*			*			4
Lin et al. (53)	*		*			*	*	*	5
Maeda (36)	*		*			*			3
Morisod et al. (52)	*		*			*	*		4
Mossetti et al. (51)	*		*			*	*	*	5
Musholt et al. (39)	*	*	*		*	*		*	6
Mutrie et al. (48)	*		*			*	*	*	5
Nakao et al. (43)	*		*			*		*	4
Ozaki et al. (37)	*		*			*	*	*	5
Pappalardo et al. (57)	*		*			*	*	*	5
Peng et al. (56)	*		*			*	*	*	5
Piazza et al. (26)	*		*			*	*	*	5
Ranganath et al. (55)			*			*			2
Segal et al. (6)	*		*			*	*	*	5
Shadmehr et al. (49)	*	*	*		*	*	*	*	7
Sharanappa et al. (66)	*		*			*	*	*	5
Su et al. (60)	*		*			*	*	*	5
Tiwari et al. (65)	*		*			*	*	*	5
Tovi et al. (33)	*		*			*	*	*	5
Tsai et al. (44)	*	*	*		*	*	*	*	7
Wada et al. (45)	*	*	*			*	*	*	6
Wang et al. (61)	*	*	*			*	*	*	6
Yang et al. (40)	*		*			*	*	*	5
Zannini et al. (38)			*			*	*	*	4

### Study Population, Perioperative Mortality, and Complications

Overall, 656 patients were included in the current systematic review. Gender of patients treated by (C)TRA was detailed in 18 papers (26, 34, 35, 38, 40, 41, 43, 46, 50–53, 55, 57, 58, 62, 64, 66) for a total of 355 patients, of whom 59% were females.

Age of patients was reported in 12 manuscripts (26, 34, 39–41, 50–53, 57, 64, 66) for a total of 121 patients, with a mean of 60 years (range, 20-85).

Seventeen manuscripts reported data on (C)TRA for DTC alone (3, 33, 34, 38, 40, 41, 43–45, 53, 58–63, 66), while 20 for mixed histologies (26, 35–37, 39, 42, 46–52, 54–57, 64, 65, 67). Overall, the distribution of histopathological types in patients treated by (C)TRA was detailed for 376 of them (59%) and their frequency in descending order was as follows: papillary (79%), follicular (7%), poorly differentiated (5%), medullary (2%), Hürtle cell (2%), anaplastic (1%), follicular variant of papillary cancer (1%), metastasis to the thyroid gland from other organs (1%), and rare histotypes such as thyroid squamous cell carcinoma, giant cell carcinoma, and carcinoma with lymphoepithelioma-like pattern (2% all together) (26, 34, 35, 37–40, 42, 44–47, 49–53, 55, 57, 62, 64, 66).

Thirteen articles exclusively described the results of (C)TRA (26, 35, 37, 38, 40, 42, 48, 50–53, 55, 57), while 24 reported data about different treatment strategies also including (C)TRA (3, 33, 34, 36, 39, 41, 43–47, 49, 54, 56, 58–67).

Eight studies did not provide detailed information on the diagnostic work-up employed for detection and assessment of airway invasion by TC (3, 33, 35–37, 42, 45, 51). The remaining 29 manuscripts specified the diagnostic methods utilized (26, 34, 38–41, 43, 44, 46–50, 52–67). Expectedly, neck and chest x-ray for airway invasion assessment were rarely mentioned in studies published after 1996. In contrast, airway endoscopy (either flexible or rigid, under local or general anesthesia) was reported in 100% of the series, computed tomography (CT) in 93%, ultrasonography (US) in 45%, and magnetic resonance (MR) in 32%.

Distinction between types of resection (purely tracheal resection and anastomosis [TRA] or also involving part of the cricoid [CTRA] with consequent thyro-crico-tracheal anastomosis) was reported in 35 papers (3, 26, 33–47, 49–58, 60–67) for a total of 619 patients undergoing 466 (75%) TRA and 153 (25%) CTRA.

Length of resection was reported in 19 papers (26, 33, 34, 37, 38, 40, 42–44, 46, 49–53, 62, 64, 65, 67), usually as range and mean in centimeters or number of removed tracheal rings (for TRA), with associated portions of adjacent cricoid cartilage (for CTRA). In some instances, detailed tables allowed to exactly know the extent of (C)TRA for each patient. However, the length of (C)TRA for 350 patients ranged between 0.5 and 6 cm (mean, 2.5).

The Shin classification was explicitly used to quantify the depth of airway invasion by TC in 10 manuscripts (26, 37, 38, 40, 41, 44, 51, 57, 62, 67), for a total of 148 patients subdivided as follows: Shin I in 12 (8%) patients, Shin II in 35 (24%), Shin III in 39 (26%), and Shin IV in 62 (42%).

**Figure 2:**
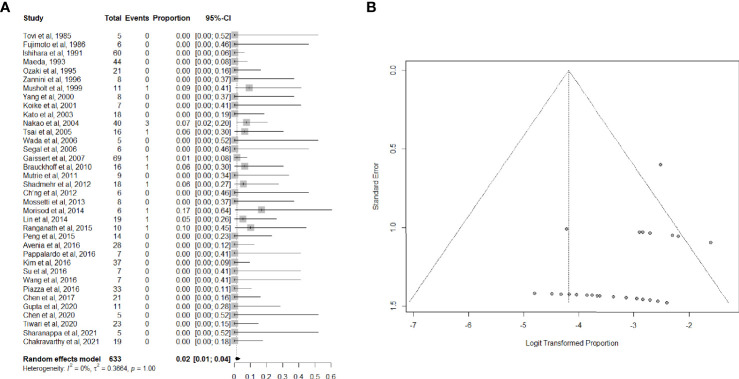
**(A)** Forest plot showing the pooled analysis of postoperative mortality and **(B)** relative funnel plot.

Data on perioperative mortality were provided in 36 articles (3, 26, 33–53, 55–67), for a total of 632 patients. The random effects model pooled risk of postoperative mortality was 2.0% (95% CI, 1.0-4.0%), with low heterogeneity. Forest and funnel plots are reported in **Figures 2A, B**.

**Figure 3:**
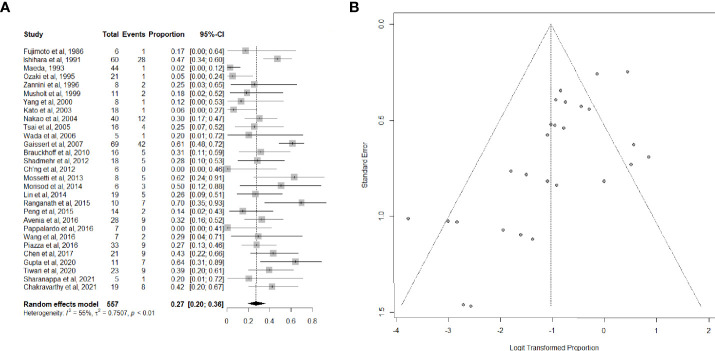
**(A)** Forest plot showing the pooled analysis of postoperative overall complications and **(B)** relative funnel plot.

Twenty-nine articles (26, 34–40, 42–47, 49–53, 55–57, 59, 61–63, 65–67), including 557 patients, provided data on the proportion of patients suffering from postoperative complications. Complications were mostly bilateral RLN palsy, anastomotic dehiscence, hypoparathyroidism, and pulmonary complications, which are listed in detail in **Table 2**. The overall summary estimate of the proportion of patients who developed any complication after (C)TRA for TC was 27.0% (95% CI, 20.0-36.0%) (**Figures 3A, B**). Heterogeneity was high (I^2 =^ 55.0%).

**Figure 4:**
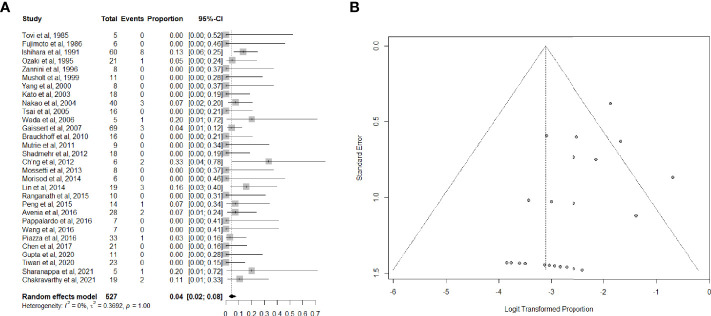
**(A)** Forest plot showing the pooled analysis of tracheotomy-dependency and **(B)** relative funnel plot.

Data on long-term tracheotomy-dependency were reported in 30 studies (26, 33–35, 37–40, 42–53, 55–57, 59, 61–63, 65–67) for a total of 527 patients. The summary estimate of the proportion of patients remaining dependent on tracheotomy after (C)TRA was 4.0% (95%CI, 2.0-8.0%). Heterogeneity of studies was low (**Figures 4A, B**).

### Oncological Outcomes and Adjuvant Treatments

Oncological outcomes details were available in 29 articles (3, 26, 34, 35, 37–41, 43–49, 51–55, 57–59, 62–66), as reported in **Table 2**. Seventeen studies (3, 26, 35, 43–49, 54, 58, 59, 62, 63, 65, 66) provided survival estimates. Five-year overall survival (OS) rate, reported by 11 studies (3, 26, 44, 48, 49, 54, 59, 62, 63, 65, 66), ranged from 61% (49) to 100% (62), whereas only 5 papers (26, 35, 43, 54, 65) reported the 10-year OS rate [ranging between 78.1% (35) and 42.1% (26)], and two studies (46, 65) also provided the 15-year OS rate (26.0% and 35.9%, respectively). Five manuscripts (26, 45, 47, 54, 58) reported 5- and 10-year disease specific survival estimates, which were in the range of 75.8-90% and 54.5-62.9%, respectively.

Specific data on adjuvant radioactive iodine (RAI) were reported by 12 papers (26, 40, 44, 46, 51–54, 62, 63, 65, 66), while 8 (26, 44, 46, 51–53, 62, 66) contained details on the use of postoperative external beam radiotherapy (EBRT). Most patients (86%; 95%CI, 61-96%; I^2^ = 22%) had been treated with adjuvant RAI, whereas indication to postoperative EBRT was far less common (11%; 95%CI, 4-30%; I^2^ = 18%). No survival analysis according to adjuvant therapies was conducted by any of the included studies.

## Discussion

### Mechanisms of Airway Invasion and Its Therapeutic Implications

When the cartilages or inter-cartilaginous ligaments are penetrated by neoplastic cells up to the level of submucosa, the TC spreads along the cartilaginous framework horizontally (following the inter-cartilaginous blood and lymphatic vessels) and vertically, before fungating into the airway lumen. As a consequence, the endoluminal real tumor extension is frequently more important than what can be seen from the outer surface of the organ (8, 19, 37). This, together with the uncertainty in the precise clinical assessment of the in-depth neoplastic extension within the cartilaginous framework, represents the most important pathological basis for justifying (C)TRA when dealing with tumors penetrating through the crico-tracheal axis, and the most evident limiting factor in supporting a window resection (*per se* based on the evaluation of the neoplastic extent just at the level of the outer airway surface).

In appropriately selected cases (i.e., for short segment airway involvement not beyond the external perichondrium) and with carefully performed surgery, shaving achieves complete resection and offers local control rates as high as 95-100% (58, 61, 68, 69). On the other hand, shaving for TC with deeper airway involvement, where R0 resection cannot be achieved or confirmed by frozen sections of cartilaginous tissues and possible microscopic penetration of the tumor into the airway submucosa *via* lymphatics piercing the inter-cartilaginous spaces may remain undetected, local failure with dismal outcomes have been reported in the literature (70, 71). Moreover, while several series included in this systematic review related to patients treated primarily by (C)TRA reported 5-year survival figures >80% (34, 41, 44, 45, 47, 48, 55, 57, 58, 62–66), the complication and mortality rates are considerably higher and survival lower when the procedure is applied as a salvage operation for recurrence after more conservative initial surgery (72, 73).

The same holds true for window resections, which are advocated by some in case of deeper tumor infiltration into the airway (20, 21). Such a surgical procedure is limited in its width and length of resection due to constraints in terms of airway stability, especially if the surgeon attempts a primary closure of the tracheal defect. As a consequence, if pedicled or revascularized myofascial/myoperichondral or skin flaps for tracheo-cutaneous fistula closure (74) are not employed, an R0 resection with negative margins by such a technique is less probable than after (C)TRA.

In case of incidental intraoperative discovery of TC invading the airway deeper than its external perichondrium, with a shaving procedure likely resulting in an R2-R1 resection, the general consensus is to convert the procedure into (C)TRA if it can be safely performed by the surgeon during the same intervention, or abort the procedure and refer the patient to a tertiary center with adequate experience in airway management (22). Clearly, in the best-case scenario, such a therapeutic option should be anticipated by performing the appropriate preoperative diagnostic work-up (including airway endoscopy and detailed cross-sectional imaging studies), referring the patient preoperatively to another team if adequate surgical expertise for (C)TRA is not available.

### Tumor Resectability and Indications for (C)TRA

Invasion of the prevertebral fascia, major cervico-mediastinal blood vessels and/or massive involvement of the thoracic trachea are situations which make TC unresectable, and are categorized as T4b (8, 75). On the other hand, the only contraindications to (C)TRA include: 1) cranio-caudal extent exceeding 5.5 cm (i.e. 11 tracheal rings or cricoid arch plus 9 tracheal rings) (76) even in young patients (while for older ones, shorter airway resections may already represent an issue, with 4 cm of length being demonstrated to significantly increase the incidence of anastomotic dehiscence) (36, 77, 78); 2) major full-thickness esophageal/hypopharyngeal involvement requiring more than shaving of the external muscular layer or limited full-thickness resection with primary closure; and 3) tumor reaching the glottic plane either anteriorly through the crico-thyroid membrane or posteriorly at the level of the crico-arytenoid joint(s) (26). All these factors should be adequately assessed in the preoperative setting, considering that the proximal and distal airway cuts should be performed one tracheal ring above and below the macroscopic invasion site as appreciated from the adventitial side and checked from inside at the level of the airway lumen, with confirmatory mucosal frozen sections as needed. Clearly, these are contraindications for (C)TRA, but such tumor extensions are amenable to more radical surgical procedures, such as pharyngo-laryngo-esophagectomy for extensive invasion of the larynx and/or esophagus. Similarly, more extensive tracheal invasion can be resected making a mediastinal tracheostomy. In such clinical scenarios, a balanced preoperative counseling that may guide patients along the difficult path of choosing between a better quality of life against a higher chance of perioperative complications should be taken into account.

Preoperative bilateral RLN palsy does not represent *per se* an absolute contraindication to (C)TRA since an R0 resection with postoperative permanent tracheotomy (and usable voice) is always better than both persistent airway disease or total laryngectomy with/without tracheoesophageal voice prosthesis insertion (26). Of note, one should consider that, in case of anterior cricoid arch resection, the ensuing bilateral loss of vocal cords tension for lack of crico-thyroid muscles, if associated with bilateral RLN palsy, may make posterior cordotomy with/without arytenoidectomy useless in the attempt of getting a patent airway without tracheostomy. Last but not least, placing a tracheostomy below the anastomotic line after (C)TRA with bilateral RLN palsy may significantly increase the risk of postoperative complications such as anastomotic dehiscence, stenosis or tracheo-innominate fistula.

Radical comprehensive approaches like (C)TRA, able to maintain a good quality of life, are strongly recommended in patients with DTC even in the presence of a limited burden of asymptomatic distant metastases (8, 26). However, general health status (age, comorbidities, compliance) and willingness to undergo surgery play a prominent role in selecting patients amenable for such a major surgical procedure.

### Role of Endoscopy and Imaging in Evaluating Shin Stage

The first endoscopic examination to be performed in every TC patient should include a flexible videolaryngoscopy, even in the absence of an appreciable hoarseness: in fact, finding a unilateral RLN palsy should prompt to the request of more targeted investigations (such as tracheoscopy and cross-sectional imaging studies) to determine and precisely evaluate potential airway involvement, both quantifying its radial (depth) and cranio-caudal extents (22). This also applies to other common signs and symptoms of advanced TC such as hemoptysis, dyspnea, dysphagia, thyroid fixation or clinically enlarged lymph nodes: even though infrequent, these findings should prompt adequate imaging to exclude aerodigestive tract invasion and quantify it for appropriate surgical treatment planning.

Transcutaneous US can detect the depth of airway invasion, reliably distinguishing superficial (Shin I-II) vs. deeper (Shin III-IV) infiltration with a diagnostic accuracy potentially reaching 93% (79–81). However, US is generally considered highly operator-dependent and less reliable for tumors larger than 4 cm or with major intralesional calcifications, as well as with significant retrosternal extension.

CT is considered superior to US and definitely more reproducible for precise three-dimensional assessment of airway invasion (22), with a mean sensitivity, specificity, and accuracy in detecting tracheal invasion of 59.1%, 91.4%, and 83.2%, respectively (82). It is important to emphasize that the CT should be performed with contrast, to give the most precise information. In particular, the most quoted CT diagnostic criteria are tumor in contact for 180° or more of tracheal circumference, deformity of the airway lumen (i.e. indentation due to pressure effect) at the level of such a contact, focal irregularity, thickening or bulging of the mucosal lining and, finally, presence of tumor within the tracheal lumen (82).

MR seems to have lower diagnostic accuracy than US and CT, with a tendency to overestimate the actual depth of airway invasion (83). Others report superior outcomes with MR compared to other imaging techniques (84). However, a tumor-airway contact exceeding 135° of the tracheal circumference seems to efficiently predict some degree of cartilaginous involvement (85).

Laryngo-tracheoscopy allows appreciation of airway invasion when the airway submucosa is reached (Shin III-IV), thus appearing as a subtle localized or diffuse mucosal redness, with elevation, edema, presence of telangiectasias and vascular engorgement, with focal erosions or endoluminal vegetations in the most obvious scenario (41). This is in line with the experience of the first author, who missed Shin II tracheal invasion in 11% of his series by endoscopy and imaging (26). The sensitivity of this tool for tracheal invasion evaluation is, in fact, reported to be around 85%, with a mean underestimation of the actual cranio-caudal tumor extent of an average of 0.8 (maximum 2) tracheal rings compared to postoperative histopathologic specimens (86).

Endobronchial US (EBUS) is the latest imaging technique for assessment of the presence and degree of airway invasion by TC. Recent reports highlight an accuracy significantly higher than those reported by CT and/or MR, with a sensitivity and specificity of 92% and 83%, respectively (84). However, EBUS is still relatively infrequently used in most medical facilities due to some inherent drawbacks such as increased invasiveness, high cost, and limited utility in evaluating tumors infiltrating at the level of the thyroid upper lobe (82).

It would therefore appear that the most adequate diagnostic algorithm for advanced TC with suspicious airway invasion should be based on careful endoscopy of the larynx and trachea, with US and subsequent CT or MR depending to the local facilities and expertise.

### Oncologic Prognosticators

Predictors of survival in advanced TC involving the airway may be patient-related (age, gender), tumor-related, and treatment-related. Among tumor-related factors, micro- vs. macroscopic extrathyroidal extension, limited to one vs. multiple organs has been recently demonstrated to play an important role (7). Strap muscles (T3b) and RLN invasion (T4a) have no prognostic influence on survival, but they do affect recurrence in contrast to laryngeal, tracheal, and esophageal involvement which heavily impact both local recurrence and survival rates (9, 87). Tracheal and esophageal invasion (T4a) present no prognostic differences when all tumor tissue can be removed within negative margins (R0 resection). By contrast, invasion of the larynx (T4a) reflects a more aggressive behavior of disease (47), even though no clear distinction is usually made in the literature with respect to the specific anatomic site(s) of TC infiltration. Intuitively, anterior cricoid involvement has a very limited impact on radicality of tumor resection and possibility of organ preservation compared to lateral and/or posterior cricoid infiltration (26). The same holds true when considering invasion of the inferior border of the thyroid laminae compared to transgression of their lateral edges in close proximity with the piriform sinus, or when dealing with a superficial (external perichondrium) vs. a full-thickness thyroid cartilage invasion.

General consensus has been reached on preserving the RLN whenever it is preoperatively functioning, even though encased by tumor, as long as it is not directly infiltrated by TC and all gross disease is removed, adding postoperative adjuvant therapy in the form of RAI or EBRT as indicated (22). Sacrifice of the RLN is generally only justified when preoperatively already non-functioning or when its preservation would inevitably leave behind gross residual disease (R2 resection) (88). In this case, RLN reinnervation by direct suture of healthy stumps at frozen sections, interposition of a nerve graft or suturing the ansa hypoglossi to the distal RLN stump may be considered to maintain vocal muscle tone and improve functional outcomes for voice rehabilitation (89–91).

Tumor histopathological type is a strong predictor of survival in TC invading the aerodigestive tract: the 5-year survival rate in DTC is around 75%, while it declines below 60% in medullary TC, and to 20% in undifferentiated tumors (26, 47).

Among procedure-related prognosticators, the most powerful seems to be R status: a number of series have confirmed better 5-year survival in R0 compared to R2 resections (90-78% vs. 50-35%) (35, 92, 93). Even R1 resections, while apparently presenting similar 5-year survivals (71, 93), in the long run are invariably associated with a higher rate of recurrence (46, 70, 71, 94). Moreover, when comparing immediate R0 resection with R1 resection followed by delayed radicalization, even considering non-organ sparing surgery, 10- and 20-year disease-free survival decrease from 67% and 50% to 7% and 0%, respectively (46). However, the absence of high-quality prospective data does not allow to solve the discrepancy between the above mentioned data and those reported by others, with no statistically significant survival differences between R0 and R1 resections (54, 61).

### Role of RAI and EBRT

It should be emphasized at the outset that postoperative RAI and/or EBRT do not replace adequate surgery with R0 resection due to the high failure rates of these adjuvant therapies in controlling residual R2 disease, especially at the level of the airway. Adjuvant therapy by RAI after (C)TRA for T4a DTC is widely used whenever sufficient iodine uptake is demonstrated. However, response to RAI often cannot be established before surgery and is not uniform especially for microscopic residual disease (R1 resection) on the airway surface (95).

Special consideration should be paid to patients who have already received RAI in the past and experience further disease progression, since tolerance to and further potential benefit from RAI is questionable in such a clinical scenario. Moreover, EBRT as initial mode of therapy in TC invading the airway deeper than its cartilaginous framework should not be offered given the limitation it places on wound healing in case of a subsequent (C)TRA (26) and the low probability of appropriate response of bulky TC invading the airway (5). Still controversial is the potential role of adjuvant EBRT after segmental R0 airway resection for DTC, especially when the laryngo-tracheal axis was the only site of macroscopic extrathyroidal extension. Since no survival analysis was available regarding the impact of both RAI and EBRT as adjuvant therapies after (C)TRA, no conclusion on their prognostic role can be withdrawn from the present systematic review.

### Study Limitations

The most notable limits of our paper are the retrospective nature and relatively low number of patients included in each case series, *per se* potentially flooded by selection biases and a wide array of geographic, therapeutic, and epidemiologic differences. Lack of details concerning the histotypes and use of adjuvant treatment protocols reduces the possibility to infer their impact on prognosis of patients treated by (C)TRA. Moreover, the evolution in the diagnostic and therapeutic strategies occurred during the long time span of our systematic review must be taken into proper consideration.

## Conclusions

The current literature is still devoid of prospective clinical trials addressing optimal management of T4a TC invading the crico-tracheal axis. However, based on the retrospective case series analyzed, even though characterized by the common biases related to the relatively small number of patients recruited in a long period of time, (C)TRA appears to be a reproducible major surgical procedure, which is able to ensure both good oncological outcomes as well as a reasonable chance of laryngeal function preservation for TC invading the trachea deeper than the level of its external perichondrium and less than 5.5 cm in length. However, the non-negligible mortality and complication rates should prompt management of these advanced tumors by skilled surgical teams in tertiary referral centers with the adequate multidisciplinary expertise, after proper diagnostic work-up and patient selection.

## Data Availability Statement

The original contributions presented in the study are included in the article/supplementary material. Further inquiries can be directed to the corresponding author.

## Author Contributions

CP, AD’C, DH, LK, GR, AR, JS, AS, RS, VV, MZ, and AF contributed to conception and design of the study. CP, DL, and MT organized the database and performed the statistical analysis. CP, DL, and MT wrote the first draft of the manuscript. CP, AD’C, DH, LK, GR, AR, JS, AS, RS, VV, MZ, and AF wrote the final draft of the manuscript. All authors contributed to the article andapproved the submitted version.

## Conflict of Interest

The authors declare that the research was conducted in the absence of any commercial or financial relationships that could be construed as a potential conflict of interest.

## Publisher’s Note

All claims expressed in this article are solely those of the authors and do not necessarily represent those of their affiliated organizations, or those of the publisher, the editors and the reviewers. Any product that may be evaluated in this article, or claim that may be made by its manufacturer, is not guaranteed or endorsed by the publisher.
